# Conserving Nervous Energy

**Published:** 1918-10

**Authors:** Edwin F. Bowers

**Affiliations:** New York City


### Conserving Nervous Energy.

By EDWIN F. BOWERS, M. D., New York City.

If human beings took as much interest in preserving their energy as they do in preserving their clothes, they would be far less interesting as medical "cases."

We waste, in a thousand useless ways, sufficient dynamic and physical force to do the work of the world many times over. We apply efficiency to about everything under the sun except ourselves.

The nervous, high-strung mother who is eternally admonishing the youngsters not to do whatever they are doing—or what about to do—is wasting her precious balance in the bank of health faster than two women could manufacture it.

And the fussy, worrying individual, who has no more idea of repose than has a Japanese waltzing mouse, is a source of anxiety to himself and a full-fledged cause of annoyance and irritation to every one who is penalized by having to associate with him.

Few realize that we have but a certain amount of physical and nervous energy to expend, and that if we waste it in futile and non-productive activities we certainly are going to run shy of "punch" when the real need comes to show efficiency. If every one could know the value of a few minutes' complete relaxation,—the sensible utility of sitting back "as loose as ashes" once in a while there would be a dearth of nervous breakdowns.

If the student, the mother, the brainworker—if every one who uses energy faster than he manufactures it—would get into the habit of "stretching out" and completely relaxing when they begin to feel fatigued, and give nature a little chance to recharge the magnetic and nervous batteries, we shouldn't need to worry about the lack of insane asylums.

These ought to be obvious facts. Perhaps we ignore them because they are obvious, and because to find out about them costs us nothing except a siege of illness or a ruction in the family. If nervous and physical conservation were something one could take in a pill, we'd be all falling over one another to get a supply.

But we spend large amounts of A-1 vitality in soul-racking worry, rapid-fire nervous explosions, and fatuous over-exertion. And then we wonder why adult longevity is decreasing. We wonder why we have to make an involuntary assignment in physical bankruptcy at forty-five or fifty, when we should have a plethoric surplus in reserve.

Not all energy dissipation, however, lies within our own preventive power. An incalculable amount of waste occurs because our eyes are too civilized—because we are overworking weakened external eye muscles sixteen hours a day too long. Nervous exhaustion, epilepsy, "stomach trouble," rheumatism—and even such grave diseases as goiter, diabetes, and Bright's disease—are cured by correcting imbalanced ocular muscles.

In the important matter of sleeping we also leave much to be desired. First, our archaic habit of sleeping two or more in bed prevents our securing the quiet and repose that should accompany any successful effort to woo Morpheus.

And we are not so particular concerning the comfort qualities of our beds, as we should be. Hitherto, anything that stood on four legs, and had a spring and a mattress, qualified as a bed—whether it fulfilled the functions of a bed or not.

Forget not that the place where we spend approximately a third of our lives has got to be something more than merely a piece of furniture, in order to contribute its quota toward maintaining our efficiency. It's got to be an institution for encouraging and facilitating sleep.

Also, we pound a lot of energy out of our systems by decorating our heels with two chunks of leather jammed full of large and very unyielding nails. We pursue the uneven tenor of our ways over hard, unsympathetic pavements, jouncing our internal organs into a state of prolapsus and mauling our sensitive backbones and delicate nervous systems at every step. A pair of springy rubber heels would take the shock out of our systems, and remove one chronic cause of irritation and debility.

Then, by wearing pointed shoes, we twist our big toes out of their true straightforwardness, and turn them in toward the foot center. This foreshortens the longitudinal arch, relaxing the tension of the muscles and ligaments forming that arch, which in turn causes a relaxation of the transverse arch muscles. This is the chief cause of flat foot, one of the meanest energy-robbers that infests the human race.

To correct flat foot, and prevent this energy loss, properly adapted, scientifically constructed shoes should be fitted and worn, and suitable exercise should be taken for a sufficient length of time—although temporarily some mechanical arch support may be required.

Anything and everything that prevents energy loss adds to the health balance. And to have a fine fat health balance is to own the highest dividend-producing investment in the Bank of Life.

---

**Acute Nephritis.** Lt. Wm. Wise of Hartford City, Ind., Ind. Med. Jour., Dec. 1917, reports a case that had 13 uraemic convulsions but that recovered.

